# Socio-Demographic, Health, and Transport-Related Factors Affecting the COVID-19 Outbreak in Myanmar: A Cross-Sectional Study

**DOI:** 10.7759/cureus.29693

**Published:** 2022-09-28

**Authors:** Khine Zin Aung, Yoshiki Kuroda, Takuji Hinoura

**Affiliations:** 1 Department of Public Health, Faculty of Medicine, University of Miyazaki, Miyazaki, JPN

**Keywords:** covid 19 retro, developing countries, contagion, myanmar, infection control measures, covid-19

## Abstract

Introduction

The coronavirus disease 2019 (COVID-19) pandemic is a worldwide threat in many aspects, making developing countries with scarce primary health care and medical services more vulnerable. Evaluation of the relationship between the COVID-19 pandemic, sociodemographic variables, and medical services provides useful information to take countermeasures to stop the infection spread and could mitigate the damage. Therefore, this study investigated the relationship between the spread of COVID-19 and sociodemographic variables, medical services, and the transportation system in Myanmar.

Methodology

This study was a cross-sectional study and was conducted using data on COVID-19 cases from August 20, 2020 to January 31, 2021 in Myanmar. We evaluated the association between the COVID-19 cases and 13 independent variables that were sociodemographic, medical services, and transportation system factors using simple linear regression analysis and multiple linear regression analysis in three phases (increasing (from August 20^th^ to October 10^th^), stable (from October 11^st^ to December 4^th^) and decreasing phases (from December 5^th^ to January 31^st^)) on the infection timeline.

Results

It was found that the population density was parallelly associated with COVID-19 cases. On the other hand, among the medical services factors, the number of doctors was parallelly associated with COVID-19 cases and the number of nurses was inversely related to COVID-19 cases.

Conclusions

The result indicated that a high population density area was a risk factor for the increase of COVID-19 cases. This supported the worldwide countermeasures to deal with the spread of the infection, such as social distancing, banning large gatherings, working from home, and implementing quarantine procedures for suspected individuals to reduce person-to-person contact. Finally, at least in Myanmar, employing a large number of nurses could reduce the emergence of new COVID-19 cases. We believe that our study can make valuable contributions to tackling future epidemics like COVID-19 not only in Myanmar but also in other developing countries.

This article was previously presented as an abstract at the 91st conference of The Japanese Society for Hygiene (JSH ) on March 08, 2021.

## Introduction

The first case of the coronavirus disease 2019 (COVID-19) was reported in Wuhan, the capital city of Hubei Province of China on December 8, 2019 [1]. The infection subsequently spread rapidly across the world, turning the epidemic into a pandemic. The World Health Organization (WHO) reported that by July 2022, over 564 million people were infected and suffering and of these nearly 6.4 million people died worldwide, while in Myanmar, the number of cumulative COVID-19 cases was 614,009 and deaths were 19,434 [2]. Coronavirus belongs to the *Coronaviridae* family, which consists of enveloped viruses with positive-sense single-stranded RNA. Primal clinical symptoms are high fever, cough, myalgia, and dyspnea, which could develop into acute respiratory distress syndrome or multiorgan failure [3], finally causing death. COVID-19 spreads from person to person via droplet infection [4]. Since the R naught (R_0_: basic reproduction number) of COVID-19 has been measured at 3.0 and above [5], it is more contagious than the influenza virus (R_0_ of Influenza A(H1N1) is 1.4 to 1.6) [6]. The average incubation period of the virus is between three and seven days, and over 80% of the virus may have been transmitted - asymptomatically or symptomatically - with the early onset of symptoms [7,8]. Since the vaccine had not yet been invented, during the early stages of the COVID-19 pandemic, countermeasures such as keeping social distancing, maintaining personal hygiene, wearing masks, and lockdowns of cities [9,10] were implemented. The transmission of the virus and the infection rate was restrained by various government policies such as mitigation and containment strategies [11].

The COVID-19 pandemic has been associated with several factors like socioeconomic factors, demographic factors, climate, and individual immunity [10,12-14], but the impact of these factors has varied across countries. Therefore, it is important to detect the factors that affected the COVID-19 infection in each area. While there has been a considerable amount of literature on the topic available with regard to developed countries, the literature is scant in developing countries, and there is none pertaining to Myanmar. Medical services are now vulnerable in Myanmar. Therefore, we tried to evaluate the relation between the COVID-19 cases and some independent variables such as sociodemographic factors, medical services, and the transportation system. We believe that our findings are a valuable contribution for formulating government policies and handling further outbreaks.

The first COVID-19 case in Myanmar was reported on March 23, 2020, and the first death was confirmed on March 31, 2020 [15]. Since the rise in patients started on August 20, 2020 and converged in February 2021, we used the COVID-19 data from August 20, 2020 to January 31, 2021.

## Materials and methods

Myanmar is divided into seven regions, seven states, and one union territory, and its total population is 51,486,253 [16]. Therefore, we set the 15 survey areas as our evaluation fields.

The number of COVID-19 cases from August 20, 2020, to January 31, 2021 was retrieved from the Ministry of Health and Sport (Myanmar) [15]. Our evaluation of independent variables in this study, such as population density; aging rate (population aged 65 years and above); unemployment rate; average monthly income; average annual temperature; number of doctors, nurses, midwives, hospitals and rural health centers; number of cars, buses, and two-wheelers were derived from the Myanmar Population Census [16] and the official government websites of Myanmar (Ministry of Labor, Employment, and Social Security [17], Ministry of Transportation and Communication [18], Ministry of Health and Sports [15], Department of Meteorology and Hydrology [19], and Myanmar Statistical Information Services [20]). The population density, aging rate, and unemployment rate were of 2014 [16]; the year 2017 was considered for the average income per month [21]; average annual temperature was based upon the years 2008-2017 [20]; medical facility and staff data was from 2021 [15]; and transportation data was from 2020 [18]. Since Myanmar is a developing country, its electronic database system is not very advance; therefore, it was very difficult to assemble independent variables data of the same year. However, we have conducted this research with limited datasets without ignoring the fact that there have been some changes in these datasets for several years.

**Figure 1:**
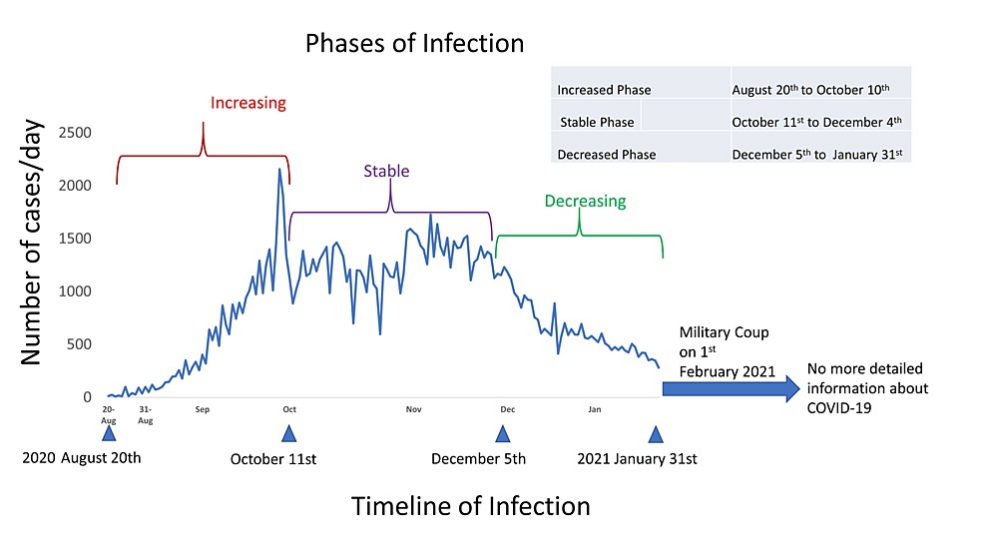
Timeline of COVID-19 infection in Myanmar Aug: August, Sep: September, Oct: October, Nov: November, Dec: December, Jan: January, COVID-19: coronavirus disease 2019

Figure 1 indicates the epidemic curve of COVID-19 cases in Myanmar. The period was divided visually into three phases as shown in Figure 1 - the increasing, the stable, and the decreasing phase - and we evaluated the relationship between the COVID-19 cases and the independent variables for each phase.

Statistical analysis

Simple linear regression analysis and multiple linear regression analysis were obtained with p < 0.05 being considered statistically significant. We used the variance inflation factor (VIF) index to evaluate the degree including multicollinearity and removed some variables to reduce the VIF index (about 5.0 or less) and to fit the model. High VIF contains much multicollinearity that needs to be corrected generally [22]. We evaluated the relation between the number of COVID-19 cases and the independent variables using multiple linear regression analysis. Excel 2003 (Microsoft Corporation, Redmond, USA) and JMP 16 (SAS Institute, Cary, USA) were used for the analysis.

## Results

Figure 1 shows the epidemic curve of COVID-19 cases in Myanmar. The number of COVID-19 cases for increasing, stable, and decreasing phases are 25668, 71983, and 42059, respectively. Table 1 summarizes the outline of the number of COVID-19 cases, the sociodemographic variables and other independent variables. Yangon is the biggest city in Myanmar and ranked first in population, population density, average income, number of doctors, and number of cars.

**Table 1:** The number of COVID-19 cases and the variables (sociodemographic, medical services and transportation system) among seven regions, seven states, and one union territory SD: standard deviation, ℃: degree centigrade, km^2^ : kilometer square

		Mean±SD(Range)
Cases of COVID-19 (/10^4^ population)		
Total cases		17.4±28.7 (1.9-118.0)
Cases in increasing phase(from August 20^th^ to October 10^th^)		2.9±7.1 (0.0-28.0)
Cases in stable phase(from October 11^st^ to December 4^th^)		8.7±16.8 (0.76-67.9)
Cases in decreasing phasephases (from December 5^th^ to January 31^st^)		6.1±5.9 (1.0-22.2)
Population density (persons/km^2^)		128.0±174.0 (13-716)
Aging rate (%)		5.5±1.1 (3.7-7.2)
Unemployed rate (%)		4.7±2.2 (2.0-10.4)
Average income (USD/month)		47.4±13.7 (23.5-71.4)
Mean annual temperature (℃）		25.6±2.8 (17.7-27.83)
Number of hospitals （/10^4^ population)		0.3±0.1 (0.2-0.6)
Number of rural health centers （/10^4^ population)		0.5±0.4 (0.1-1.8)
Number of Doctors （/10^4^ population)		1.7±1.4 (0.7-5.3)
Number of nurses （/10^4^ population)		5.2±3.2 (2.6-11.2)
Number of midwives (/10^4^ population)		5.1±2.7 (3.3-12.8)
Number of two wheeled vehicles （/10^4^ population)		1072.3±1021.9 (69.8-3356.6)
Number of cars （/10^4^ population)		66.7±125.8 (2.4-481.3)
Number of buses （/10^4^ population)		6.8±14.4 (0.5-57.0)

We separately evaluated the correlation between the number of COVID-19 cases (per 10,000 people) and the variables in each of the three phases by using a simple linear regression analysis (Table 2) in each phase. The results indicated a parallel relationship between COVID-19 cases and population density, average income, and the number of doctors, cars, and buses.

**Table 2:** The correlation between COVID-19 cases and the variables (sociodemographic, medical services and transportation system) *: P<0.05, **: P<0.01, CC: Correlation Coefficient

		Phase		
	Total cases (CC)	Increasing (CC)	Stable (CC)	Decreasing (CC)
Population density (persons/km^2^)	0.973**	0.935**	0.962**	0.878**
Aging rate (%)	0.178	0.128	0.157	0.267
Unemployed rate (%)	-0.005	0.100	0.002	-0.150
Average income (USD/month)	0.580*	0.460	0.551*	0.700**
Mean annual temperature (℃）	0.256	0.219	0.253	0.262
Number of Hospitals （/10^4^ population)	0.107	-0.390	-0.415	-0.457
Number of rural health centers （/10^4^ population)	-0.323	-0.290	-0.316	-0.326
Number of doctors （/10^4^ population)	0.689*	0.624*	0.649**	0.754**
Number of nurses （/10^4^ population)	-0.082	-0.068	-0.091	-0.057
Number of midwives (/10^4^ population)	-0.133	-0.109	-0.125	-0.160
Number of two wheeled vehicles （/10^4^ population)	-0.072	-0.204	-0.129	0.262
Number of cars （/10^4^ population)	0.927**	0.875**	0.907**	0.880**
Number of buses （/10^4^ population)	0.957**	0.946**	0.953**	0.795**

Additionally, we verified the relationship between the number of COVID-19 cases and the variables using multiple linear regression analysis (Table 3, Model 1). Thereafter, we eliminated the number of cars, buses, nurses, midwives, and hospitals from the variables to minimize VIF (about 5.0 or less) for reducing multicollinearity, and re-evaluated the relationship (Table 3, Model 2). We detected a statistically significant parallel relationship between the cases and population density. However, this relationship was not observed for the decreasing phase.

**Table 3:** The relation between COVID-19 cases and the variables (sociodemographic, medical services and transportation system) by multiple linear regression analysis *: P<0.05,  **: P<0.01,  β:Standardized partial regression coefficient, VIF: Variance Inflation Factor Model 1: slot all variables in multiple linear regression analysis; Model 2: remove number of cars, buses, nurses, midwives, hospitals to hold VIF under 10

			Phase							Phase			
		Total (β)	Increasing (β)	Stable (β)	Decreasing (β)	VIF			Total (β)	Increasing (β)	Stable (β)	Decreasing (β)	VIF
Population density (persons/km^2^)	Model 1	0.10	0.94	0.11	-0.95	371.1		Model 2	0.93**	0.93**	0.99**	0.59	4.9
Aging rate (%)		0.04	0.18	0.14	-0.44	28.0			-0.01	-0.07	-0.06	0.19	2.2
Unemployed rate (%)		0.15	0.22	0.08	0.24	7.0			0.09	0.18	0.07	0.04	1.7
Average income (USD/month)		-0.12	0.06	-0.03	-0.58	19.7			0.13	-0.02	0.12	0.31	3.5
Mean annual temperature (℃）		0.11	-0.35	0.17	0.48	16.4			-0.21	-0.18	-0.14	-0.39	4.8
Number of two wheeled vehicles （/10^4^ population)		0.00	-0.14	-0.06	0.32	33.8			-0.09	-0.17	-0.13	0.12	2.1
Number of cars (/10^4^ population)		0.82	-0.52	1.09	1.53	189.3			―	―	―	―	―
Number of buses (/10^4^ population)		-0.02	0.76	0.14*	-1.41	281.6			―	―	―	―	―
Number of rural health centers (/10^4^ population)		-1.00	-0.09	-0.67	-2.83	289.4			-0.03	-0.08	0.03	-0.13	4.5
Number doctors （/10^4^ population)		0.09	0.15	-0.45	1.55	209.8			0.06	0.12	-0.02	0.20	5.3
Number of nurses （/10^4^ population)		-0.70	0.05	-0.22	-2.86	398.0			―	―	―	―	―
Number of midwives (/10^4^ population)		1.50	-0.92	0.93	5.76	1251.3			―	―	―	―	―
Number of hospitals （/10^4^ population)		0.10	0.99	0.23	-1.33	389.7			―	―	―	―	―

After we divided the variables into two categories - “the variables excluding medical services” and “the variables concerning medical services”, we also investigated the relationship between the number of COVID-19 cases and the variables (Tables 4, 5 ) while excluding the number of cars, buses, midwives, and hospitals to minimize VIF. The results showed that the population density was statistically significantly associated with increased cases during all three phases (increasing, stable, and decreasing) (Table 4 Model 4). The number of doctors displayed a significant parallel association, while the number of nurses revealed a significant reverse association statistically (Table 5 Model 6).

**Table 4:** The relation between COVID-19 cases and the variables excluding medical service by multiple linear regression analysis *: P<0.05, **: P<0.01,  β: Standardized partial regression coefficient, VIF: Variance Inflation Factor Model 3: slot all variables in multiple linear regression analysis; Model 4: remove the number of cars and buses to hold VIF under 10.

			Phase							phase			
		Total(β)	Increasing (β)	Stable (β)	Decreasing (β)	VIF			Total (β)	Increasing (β)	Stable (β)	Decreasing (β)	VIF
Population density (persons/km^2^)	Model 3	0.57	0.39	0.54	0.79	23.2		Model 4	0.97**	1.02**	0.97**	0.75**	1.8
Aging rate (%)		0.05	0.02	0.03	0.13	2.6			-0.03	-0.11	-0.05	0.12	1.6
Unemployed rate (%)		0.10	0.19*	0.08	0.00	1.6			0.09	0.17	0.07	0.02	1.6
Average income (USD/month)		0.13	0.04	0.13	0.20	3.9			0.14	0.00	0.11	0.35	3.3
Mean annual temperature (℃）		-0.17	-0.13	-0.16	-0.22	2.5			-0.19	-0.13	-0.16	-0.31	2.2
Number of two wheeled vehicles （/10^4^ population)		-0.06	-0.06	-0.09	0.05	1.9			-0.08	-0.14	-0.13	0.2	1.4
Number of cars (/10^4^ population)		0.05	-0.28	-0.14	1.01	24.9			―	―	―	―	―
Number of buses (/10^4^ population)		0.35	0.89*	0.55	-0.95	35.7			―	―	―	―	―

**Table 5:** The relation between COVID-19 cases and the variables concerning medical service by multiple liner regression analysis *: P<0.05, **: P<0.01,  β: Standardized partial regression coefficient, VIF: Variance Inflation Factor Model 5: slot all variables in multiple linear regression analysis; Model 6: remove the number of midwives and hospitals to hold VIF under 10.

			Phase							Phase			
		Total(β)	Increasing (β)	Stable (β)	Decreasing (β)	VIF			Total(β)	Increasing (β)	Stable (β)	Decreasing (β)	VIF
Number of rural health centers (/10^4^ population)	Model 5	‐1.60*	-1.72	‐1.82*	-0.55	26.1		Model 6	0.44	0.39	0.42	0.48	3.6
Number doctors （/10^4^ population)		0.60	0.32	0.54	1.01*	14.0			1.15**	1.04**	1.10**	1.24**	2.3
Number of nurses （/10^4^ population)		-0.99	-0.68	-1.04	-1.06	25.8			‐0.92*	-0.81	‐0.89*	‐0.96**	4.5
Number of midwives (/10^4^ population)		2.70**	2.90*	2.93**	1.32	47.2			―	―	―	―	―
Number of hospitals （/10^4^ population)		-0.60	-0.91	-0.57	-0.21	44.3			―	―	―	―	―

## Discussion

The COVID-19 pandemic has caused immense suffering and many deaths worldwide. This disease has had a detrimental impact globally and affected both developed and developing countries, including Myanmar. Immunization by vaccination was limited to developed countries, and it took a long time for the vaccine to be available in developing countries. Therefore, it is important to understand the risk factors that can cause the infection to spread, and to put into place effective countermeasures.

Since the coronavirus gets transmitted from person to person through droplet infection, contact with people is a high-risk factor. Living in urban or major cities [10,23] and a crowded public transportation system [24-26] could be assumed risk factors for increasing COVID-19 cases. It was reported that there was a parallel relation between population density and virus contagion and morbidity [27,28]. Moreover, the number of buses was considered a more important factor for rapid contagion than the number of cars [24,26,29,30]. Using public transportation could increase the risk of contagion [24,26,29,30]. Other factors such as high unemployment rate [14], being senior citizens [31], and residing in areas with poor medical facilities [32,33] could be factors causing the rise of COVID-19 cases. Additionally, high income, a developing economy, and high employment rate could induce the rapid spread of emerging infectious diseases due to increased human mobility necessitated by economic activity [14,34]. Therefore, it is important to detect risk factors for the increase of COVID-19 cases, and prevent the infection when effective vaccines are not available. We evaluated the relationship between the COVID-19 cases and the variables such as sociodemographic and other factors (population density, aging rate, unemployment rate, average income per month, average annual temperature, numbers of hospitals, health centers, doctors, nurses, and transportation system (buses, cars and two-wheelers)).

Table 2 indicates that population density, average income per month, and the number of doctors, cars, and buses had a parallel correlation with the number of COVID-19 cases. Our results aligned with those of other studies [24-28,35,36].

We also re-evaluated the relationship using multiple linear regression analysis to evaluate the influence of the variables individually with reducing multicollinearity. We found that only population density was significantly associated with the increased number of COVID-19 cases (Table 3 Model 2). It means that living in urban or major cities could be a potential risk for the infection spread. Therefore, staying at home, keeping social distance, and banning large gatherings could be effective countermeasures to contain the spread of the COVID-19 infection, especially for those living in densely populated areas. During the decreasing phase, the population density was not associated with the increase in COVID-19 cases. Though the reason is unclear, we believe that the preventive countermeasures were already effective in the decreasing phase. We could not evaluate the relation between the COVID-19 cases and the transportation system, such as the number of cars and buses, because of high multicollinearity with other variables.

Some references suggest that medical services were a mitigating factor in containing the spread of COVID-19 [33,37,38]. However, we could not evaluate this due to high multicollinearity with other variables. Therefore, we evaluated the relation between the COVID-19 cases and “the variables excluding medical services” and “the variables concerning medical services” (Tables 4, 5). Our finding was that there was a significant parallel correlation between the population density and the number of doctors with the number of COVID-19 cases, while there was an inverse correlation with the number of nurses. Some studies showed that medical services could be an important protective measure for COVID-19 infection [33,37,38]. However, the relation between the number of doctors and the number of COVID-19 cases in our study did not align with the results of other studies [33,39]. A plausible reason is that we detected a high correlation between the population density and the number of doctors. First, there are not many doctors in Myanmar, and they are concentrated in the highly populated areas; moreover, their main role is that of curative care. The infection prevention activities are mainly carried out by nurses. Previous studies have also indicated that nurses played an important role in the successful prevention and control of mosquito-borne outbreaks, such as the zika and dengue viruses [40]. The results of our study did not show a correlation between the number of nurses and the population density. Notably, there is an imbalance between doctors and nurses in Myanmar [41].

Some studies have reported that high incomes and increasing employment rates are factors responsible for the spread of emerging infectious diseases [14,34]. Additionally, elderly people are more prone to infection [31] and could be a risk factor for contagion. However, we could not find any significant association between the COVID-19 cases and the aging rate, unemployment rate, or average income in Myanmar. Though we could not provide sufficient reasons for this, the difference in the unemployment rate, average income and aging rate between areas was small (Table 1) when compared to other reports [42].

Our study is not without limitations. We acknowledge that vaccination is an important countermeasure for infectious diseases. However, since most people were not vaccinated before January 31, 2021, we could not evaluate the influence of vaccination on the spread of the infection. Moreover, Myanmar was faced with a military coup on February 1, 2021, which hindered our efforts to obtain additional detailed information on the COVID-19 contagion and vaccination status.

The formulation of governmental policies with regard to COVID-19 is an important countermeasure for reducing infection. The countermeasures employed in Myanmar were social distancing, restricting gatherings of more than 15 people, a temporary ban on international commercial flight landings, establishing public health labs, and home quarantining, which isolated infected individuals. However, the Myanmar government was unable to systematically implement these countermeasures, and it was difficult to obtain the data regarding state- and region-wise anti-COVID-19 infection policies. Therefore, we could not evaluate the relation between the number of COVID-19 cases and the policies.

In general, using the latest data that are also of the same year is important for high validity. Myanmar is a developing country, and its electronic database is still not fully developed. Additionally, the political situation in Myanmar is currently unstable. The latest national demographic survey (census) was carried out in 2015, and the largest national survey, Myanmar Living Conditions Survey, was carried out in 2017. It is impossible to obtain the data for the same year as the timeline of COVID-19 outbreak. Therefore, this study had to be carried out with limited valuable data. Additionally, it is essential to identify the difference in the variables mediating the COVID-19 cases between urban and rural areas. However, unfortunately, we could not obtain detailed data on COVID-19 cases and variables area-wise (urban and rural).

Despite the limitations mentioned above, this research was the first report concerning the relationship between COVID-19 cases and variables, such as sociodemographics and other factors. We believe that this report could help to formulate countermeasures in Myanmar if and when confronted with an epidemic in the future.

## Conclusions

This study was conducted to evaluate the relationship between the COVID-19 cases and the variables concerning the sociodemographic, medical, and transportation systems. We identified population density to be a contributing factor to the spread of infection and the number of nurses as a protective factor, in Myanmar. However, we could not indicate any correlation between the COVID-19 cases and aging rate, unemployment rate, and average income, unlike other previous studies. This is the first study to investigate the various factors regarding the COVID-19 contagion in Myanmar. It aims to provide useful information to control the spread of infectious diseases like COVID-19 and makes valuable contributions for policy-makers to consider in times of future epidemics not only in Myanmar but also in other developing countries.
